# Effect of In-Vehicle Audio Warning System on Driver’s Speed Control Performance in Transition Zones from Rural Areas to Urban Areas

**DOI:** 10.3390/ijerph13070634

**Published:** 2016-06-25

**Authors:** Xuedong Yan, Jiali Wang, Jiawei Wu

**Affiliations:** 1 MOE Key Laboratory for Urban Transportation Complex Systems Theory and Technology, Beijing Jiaotong University, Beijing 100044, China; 15120881@bjtu.edu.cn; 2 Department of Civil Environmental Construction Engineering, University of Central Florida, 4000 Central Florida Blvd., Orlando, FL 32816, USA; wjw345178371@gmail.com

**Keywords:** transition zones, in-vehicle audio warning system, traffic calming measures, driving simulator, driving behavior

## Abstract

Speeding is a major contributing factor to traffic crashes and frequently happens in areas where there is a mutation in speed limits, such as the transition zones that connect urban areas from rural areas. The purpose of this study is to investigate the effects of an in-vehicle audio warning system and lit speed limit sign on preventing drivers’ speeding behavior in transition zones. A high-fidelity driving simulator was used to establish a roadway network with the transition zone. A total of 41 participants were recruited for this experiment, and the driving speed performance data were collected from the simulator. The experimental results display that the implementation of the audio warning system could significantly reduce drivers’ operating speed before they entered the urban area, while the lit speed limit sign had a minimal effect on improving the drivers’ speed control performance. Without consideration of different types of speed limit signs, it is found that male drivers generally had a higher operating speed both upstream and in the transition zones and have a larger maximum deceleration for speed reduction than female drivers. Moreover, the drivers who had medium-level driving experience had the higher operating speed and were more likely to have speeding behaviors in the transition zones than those who had low-level and high-level driving experience in the transition zones.

## 1. Introduction

Speeding is one of the most common driving behaviors and is also considered one of the largest contributors to road injuries and fatalities [1,2,3]. The recent statistics from the Chinese Road Traffic Statistics Yearbook has reported that a total of 8812 speeding-related traffic fatalities happened in 2011, which accounted for 14.13% of all traffic deaths [4]. In the United States, 32,719 traffic fatalities occurred in 2013, and among them, 9613 (29%) deaths were related to speeding, which resulted in $40.4 billion of annual economic cost to society [5]. As the speed of the vehicle increases, the stopping distance increases, and the response time decreases, which enable dangerous situations [1]. Furthermore, the increased speed also results in difficulty in lane control on curve segments and a reduction in friction between the tires and the road [6]. Therefore, even small increases in speed could increase the probability of car accidents and the severity of the traffic crash, particularly in crashes with pedestrians and cyclists [7,8,9,10]. In addition, a higher driving speed would also contribute to the larger collision energy between vehicles, road users or obstacles [11].

Speeding frequently happens in areas where there is a mutation in speed limits. One of the areas connects a road section with a higher posted speed limit to one with a lower speed limit. The drivers are expected to lower their operating speed in this area. These road segments are called transition zones that usually exist in the entrance to an urban area [12,13]. When drivers enter the transition zone, they tend to underestimate their speeds because of long periods of high speed driving, which is called speed adaptation [14,15,16]. Therefore, drivers are still in high-speed driving where traffic density, pedestrians, cyclists and virtual complexity increase, which leads to a higher crash rate in the transition zones [17,18]. The crash data in Victoria, Australia, in 1992 indicated that the fatality rate (45 fatalities per 100 million vehicle-miles per year) in transition zones was significantly higher than that (27 fatalities per 100 million vehicle-mile per year) in rural zones [19].

During the past thirty years, a number of studies have been conducted to improve the safety in transition zones. Typically, four types of measures that have been implemented in transition zones include geometric design, traffic control devices, surface measures and roadside features [12]. The effective measures of geometric design mainly include central islands [20], narrowing roads [21], road diets [22,23,24,25], etc. Charlton and Baas [20] found that median islands and visual chicanes reduced speeds respectively by about 9% and 26%. Based on a driving simulator study, it was found that after implementing lane narrowing measures through using painted center islands and edge markings, the 85th percentile speed was lowered by 5.5 km/h [21]. Knapp and Rosales [24] reported that the road diets could result in speed reductions of 8 km/h or less, but up to a 70% reduction in excessive speeding. Additionally, the road diets could contribute to as high as 44% crash reductions [25]. The traffic control devices include countdown speed signs [26], speed feedback signs [27], removal of pavement markings [28], etc. The driving simulation results showed that the countdown signs produced a 9% speed reduction in the scenario of approaching a village [26]. Donnell and Cruzado [27] conducted the research on the use of speed feedback signs in Pennsylvania, indicating a mean speed reduction of approximately 10 km/h. However, the effect was achieved only when the sign was in place, but after the sign was removed, the mean speed rebounded to the levels before. The removal of the directional dividing lines could contribute to an 11-km/h speed reduction and a 5% reduction in travel speeds [28]. The road surface measures include vertical deflections (e.g., speed humps and raised intersections) [20] and rumble-wave surfaces [29]. However, vertical deflections at high-to-low speed transitions have not been widely used in practice, since these may have the potential for vehicle damage. Even worse, the vehicle may have loss of control crashes because of the vertical deflection [12]. Nonetheless, Charlton and Baas [20] reported that speed cushions and speed humps in transition zones could reduce speeds by 9% and 21%, respectively. The U.K. Department for Transport [29] tested a quieter rumble-wave surface and found that the mean and the 85th percentile speeds were reduced by 2% and 1% in transition zones, respectively. The treatment of roadside features is to put measures or elements at the urban/rural threshold and present a visual cue to drivers, showing that this is the point of change in roadway character [12]. Andersson et al. [30] analyzed the safety performance of 251 town gates in transition zones in Denmark. Three to five years before-after crash data were collected to estimate the safety performance. The results indicated that the town gates had a positive effect on reducing the crashes in transition zones. In addition, it was also found that combining physical and visual treatments provided the best safety performances. Veneziano et al. [31] examined the crash data collected before and after gateway constructions areas in California and came to the conclusion that the gateway constructions did not contribute to the traffic crashes.

Although the traditional engineering countermeasures listed above have been applied and tested for speed reduction in transition zones either in field or lab environments, a few studies have been conducted to use the intelligent transport technologies to solve this problem. The Intelligent Speed Adaptation (ISA) system is capable of monitoring the driving speed and giving feedback to the drivers in a timelier manner, compared to the prescribed (fixed or variable) limits. While it could improve the driving behavior with respect to speeding and traffic safety [32,33,34], more and deeper studies are needed. In recent years, more efforts have been made to develop the in-vehicle warning system in order to improve drivers’ vehicle control performance, especially in dangerous driving environments. The in-vehicle warning system has been widely deployed to help drivers in detecting traffic hazards, such as red-light running (RLR) violations and reminding drivers of the highway work zones ahead. According to the experimental results, the in-vehicle warning system can dramatically decrease RLR violations and has a significantly better effect than the engineering RLR countermeasures [35,36]. When using the in-vehicle warning system in work zones, the previous studies mostly focused on the impacts of drivers’ demographical factors (e.g., gender, education, driving experience and age) on their speed patterns and the effect of enhancing the traffic safety in the work zones. The results indicated that the drivers’ socio-demographic factors could not significantly affect their speed patterns, but the driving performance, such as mean driving speeds, speed variance and deceleration, would be further investigated either in simulation environments or on real roads [37,38,39]. Liu et al. also mentioned that drivers’ safety could benefit substantially from the technological advances in in-vehicle warning information systems. It was found that that drivers’ safety could benefit substantially from the technological advances in in-vehicle warning information systems [40]. In transition zones, the in-vehicle warning information system would also have a large potential for reminding drivers to lower their speeds to a safe range before entering into the city. When drivers are approaching the lower speed limit sign, the vehicles’ speeds can be dynamically measured with the sensors equipped in vehicles, such as GPS. If the vehicle’s speed exceeded the speed limit, the in-vehicle device would activate the audio speeding warning message. Thus, the system would remind drivers to lower their speed immediately and further avoid potential crashes because of speeding.

The purpose of this study is to investigate the impacts of the in-vehicle audio speeding warning system on drivers’ speed performance in transition zones based on driving simulator experiments. Additionally, the lit speed limit sign is chosen as the traditional engineering countermeasure. Therefore, four scenarios, including the baseline scenario, the scenario with the lit speed limit sign, the scenario with in-vehicle audio speeding warning message and the scenario with a combination of the speeding warning message and the lit sign, were built in the driving simulator to examine the effects of the in-vehicle audio speeding warning message system on drivers’ speed control performance in transition zones, compared to the traditional engineering countermeasures. The speed control performance measures included the speed profile, speeding ratio and deceleration pattern.

## 2. Materials and Methods

### 2.1. Subjects

The experiment was a 4 (scenario) × 2 (gender) × 3 (driving experience) within-subject repeated measures design. A total of 41 participants in two gender groups, 21 male drivers and 20 female drivers, completed the driving simulator experiment without any motion sickness. All of the participants were local drivers in Beijing with a valid driving license and had driving experience for at least one year. The average age of the participants was 32.7 ranging from 25–50 years old, with a standard deviation of 6.9 years. The participants’ average self-reported total driving mileage was 11.5 km, with a standard deviation of 12.3 km. Since the total mileage could reflect drivers’ driving experience, the participants were allocated into three groups based on the total mileage. The participants whose total mileages were less than 30 thousand kilometers were defined as the low-level driving experience drivers. The participants whose total mileages were between 30 and 100 thousand kilometers were in the medium-level driving experience group. Other participants whose total mileages were more than 100 thousand kilometers were assigned into the high-level driving experience group. Finally, there were 14 participants (7 males vs. 7 females) in the low-level driving experience group, 14 participants (7 males vs. 7 females) in the medium-level driving experience group and 13 participants (7 males vs. 6 females) in the high-level driving experience group.

### 2.2. Apparatus/Equipment

**Figure 1:**
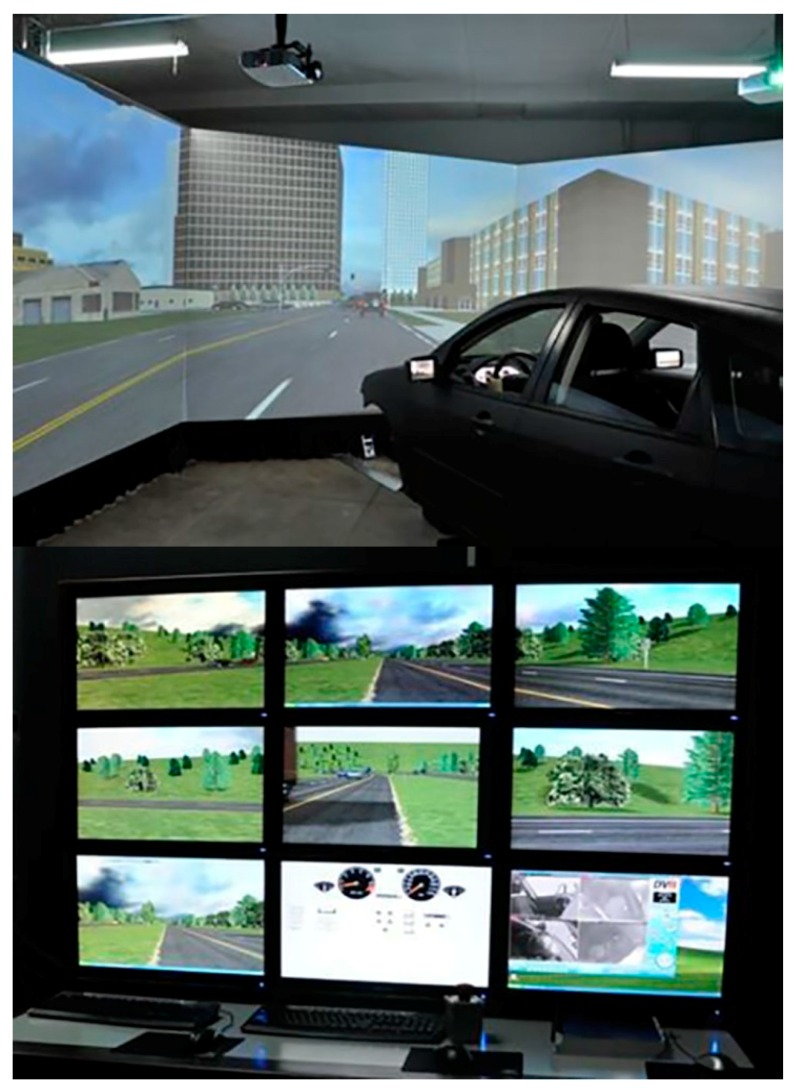
The Beijing Jiaotong University (BJTU) driving simulation system.

This study used the Beijing Jiaotong University (BJTU) driving simulator as a tool for the experiment and data collection, as shown in Figure 1. The hardware of the simulator mainly includes the full-size cabin, the two degrees of freedom dynamic simulation platform, the visual simulation system, the mimetic environmental noise and vibration simulation system, the digital video reproduction system, the vehicle dynamics simulation system and the surveillance system. The full-size cabin is identical to a real vehicle (a Ford Focus) with real operation interface. The software, including Simvista and Simcreator, can be applied by researchers to create driving scenarios with virtual traffic environments and the virtual road networks. The data sampling frequency was 60 Hz.

### 2.3. Scenario Design

**Figure 2:**
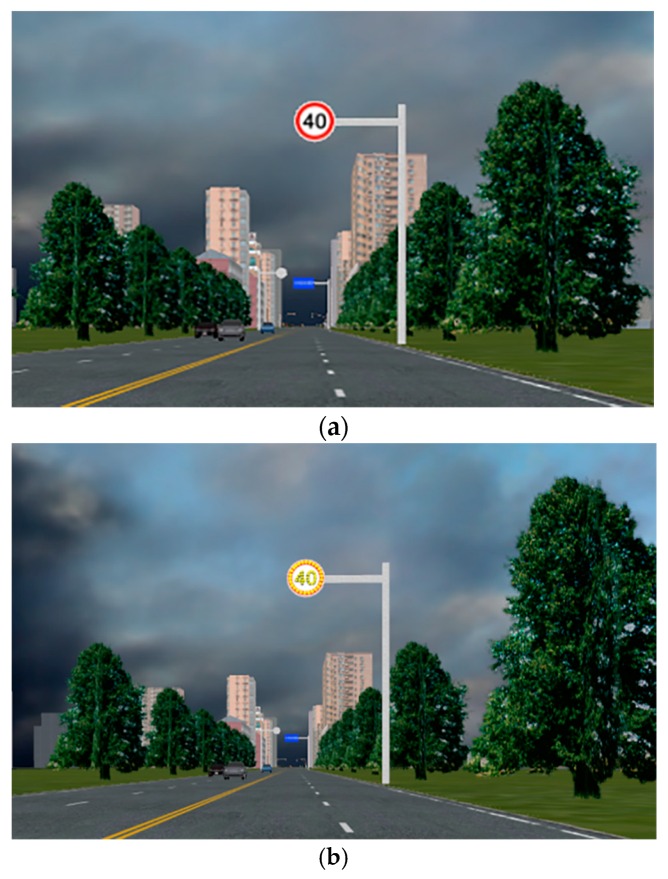
Snapshots of the traditional and lit speed limit signs. (**a**) The traditional speed limit sign; (**b**) the lit speed limit sign.

In order to investigate the effectiveness of different countermeasures on reducing speed in transition zones, a simple road network was established in the driving simulator, which was composed of four-lane road segments in both rural and urban areas. The road in rural areas was 3000 m and the speed limit 80 km/h, while the road in urban areas was 600 m, and the speed limit of the urban street was 40 km/h. According to the literature [41], the transition zone was designed as 400 meters long, which connected the rural area and the urban area. Four different scenarios were tested in the driving simulator. The first scenario was the baseline scenario, which only included a traditional 40 km/h speed limit sign in the transition zone. The speed limit sign was located at 320 m before the urban area, as shown in Figure 2a. Since there are no specific standards applied to the distance between the speed limit sign and the city edge, 320 m was designed as a buffer zone for drivers’ further reducing their operating speed, especially when the in-vehicle audio warning system was used to remind the speeding drivers. In the second scenario, the traditional speed limit sign was replaced by the lit speed limit sign, which was shown in Figure 2b. In the third scenario, the audio warning system was established in the driving simulator, and the speed limit sign was the traditional one. The audio warning system, which was triggered at the point of the speed limit sign, would play different audio speeding warning messages based on the vehicle’s spot speed. For example, when the speed was under 40 km/h at the point of the speed limit sign, the audio warning message would read “The city is ahead and speed limit is 40 km/h. Please pay attention to driving safety.” When the speed was between 40 km/h and 45 km/h, the audio warning message would read “The city is ahead and speed limit is 40 km/h. Please slow down.” When the speed was between 45 km/h and 50 km/h, the audio warning message would read “The city is ahead and speed limit is 40 km/h. You have been speeding by 20%. Please slow down as soon as possible.” When the speed was between 50 km/h and 60 km/h, the audio warning message would read “The city is ahead and speed limit is 40 km/h. You have been speeding by 30%. Please slow down as soon as possible.” When the speed was between 60 km/h and 70 km/h, the audio warning message would read “The city is ahead and speed limit is 40 km/h. You have been speeding by 50%. Please slow down as soon as possible.” When the speed was over 70 km/h, the audio warning message would read “The city is ahead and speed limit is 40 km/h. You have been excessively speeding. Please slow down at once.” According to the design and results, the warning messages were about 4–7 s long, and the test drivers traveled about 40–100 m during the warning message delivery. Finally, the fourth scenario combined the lit speed limit sign and the audio warning system together.

Moreover, to make the driving scenarios seem more realistic, a number of vehicles were designed in the road network. However, there were no other vehicles in front of the simulator vehicle before drivers entered the transition zone, so that the driving speed was dependent on the drivers themselves.

### 2.4. Experiment Procedure

Before the experiment, each participant was asked to read and sign an informed consent form (per the IRB). In addition, all participants were required to fill out a personal information form and take a short training session. The participants were advised to drive safely and follow the traffic laws in real-life situations. They were also told that they could quit the experiment at any time in case of driving simulation sickness or any kind of discomfort in the training session. The participants were trained to familiarize themselves with the operation of the driving simulator for at least 5 minutes, which included straight driving, acceleration, deceleration, left/right turn and other basic driving behaviors. Then, they performed the formal experiments with the four test scenarios in a random sequence to eliminate any order effects, and a break of at least 5 min was allowed between the tests.

### 2.5. Dependent Measures

Each participant completed the four scenarios in the driving simulator, and 164 records of data were collected. Several key variables that could reflect drivers’ speed control performance were extracted from the raw data. The related dependent measures for further analyses were defined as follows:

■ Operating speed (km/h): the participant vehicle’s operating speed for every 100 m before the city entrance.
■ Entrance speed (km/h): the participant vehicle’s operating speed at the city entrance.
■ Speeding ratio (%): the percentage of drivers who were speeding over the 40 km/h speed limit at the city entrance.
■ The maximum deceleration (m/s^2^) and the corresponding location (m): the maximum deceleration during the drivers’ deceleration period in the transition zone and the corresponding location.
■ The average deceleration (m/s^2^): the average deceleration during the drivers’ deceleration period in the transition zone.

## 3. Results and Analyses

### 3.1. Speed Profile Analyses

The basic statistical descriptions of average operating speed for different scenarios, gender and driving experience at different locations are listed in Table 1. The location of 0 m refers to the position of the 40-km/h speed limit sign. The location of the negative values and positive values stand for the distance before the speed limit sign and after the speed limit sign, respectively. From Table 1, it is found that the average operating speed of male drivers is higher than that of female drivers at each location, indicating that females have a lower traveling speed compared to the male drivers. This finding is consistent with Kennedy’s results (2005) [42]. For the driving experience, the results show that drivers who have medium-level driving experience have the highest operating speed at each location. Moreover, the ANOVA results also indicate that there is a significant difference between genders (*p* < 0.001) and driving experience (*p* < 0.001).

In order to analyze the impacts of different countermeasures on speed reduction at different locations, the paired *t*-test was conducted between the baseline (first scenario) and the other three countermeasures (second, third and fourth scenario), as shown in Table 2. For the operating speed before the speed limit, the *p*-values of the paired *t*-test are all higher than 0.05, indicating that there is an insignificant difference between the baseline and the other three countermeasures. However, at the location of 0 m and 100 m, the operating speeds in the fourth scenario (lighting + audio warning) are obviously lower than in the first scenario (0 m: *p*-value = 0.002; 100 m: *p*-value = 0.003). The results imply that only when both the lit reminder speed limit sign and the audio speeding warning messages are applied in the scenario did the drivers’ speeds significantly decrease at the location of 0 m and 100 m. Moreover, the operating speeds in the third scenario and fourth scenario are statistically lower than the baseline scenario at the location of 200 m and 300 m, which indicates that as long as the audio warning system is applied in the vehicle, the drivers’ speeds can be significantly reduced at the location of 200 m and 300 m. Overall, if the lit speed limit sign is the only countermeasure in the scenario, the drivers’ operating speed would remain the same. In other words, the lit speed limit sign does not affect the drivers’ speeds in the transition zone. 

Then, entrance speed was also collected, since the fundamental purpose of implementing traffic calming measures is to make drivers reduce their speeds to the safe range when they enter the urban area. For all of the subjects, the average speed at the city entrance is 41.85 km/h with a standard deviation of 8.61 km/h. Table 2 indicates that compared to the baseline scenario, both the audio warning scenario and the combination of the audio warning and lit speed limit sign could effectively lower the operating speeds at the city entrance. Furthermore, the ANOVA test for entrance speed indicates that there is no significant difference between male and female drivers (M = 43.29 km/h, SD = 9.63 km/h vs. M = 40.33 km/h, SD = 7.14 km/h; *p* = 0.113). However, it is found that the driving experience is the significant factor that affects the entrance speed (*p* = 0.029). The result shows that the drivers with medium-level experience have higher entrance speeds than the other two levels. 

### 3.2. Speeding Ratio Analysis

**Figure 3:**
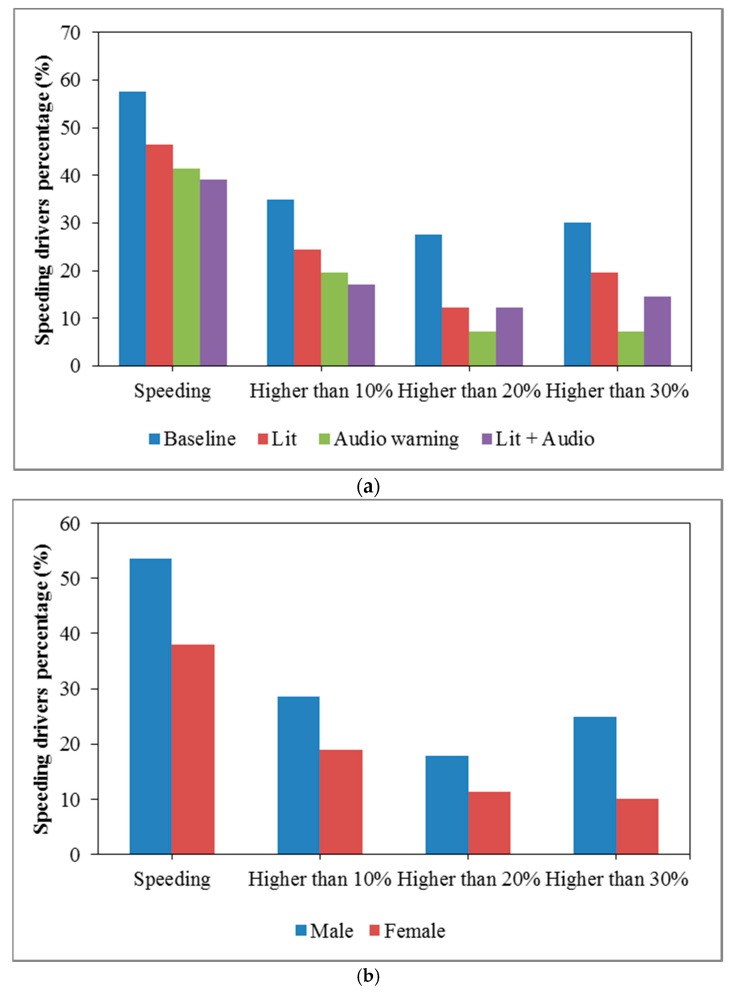

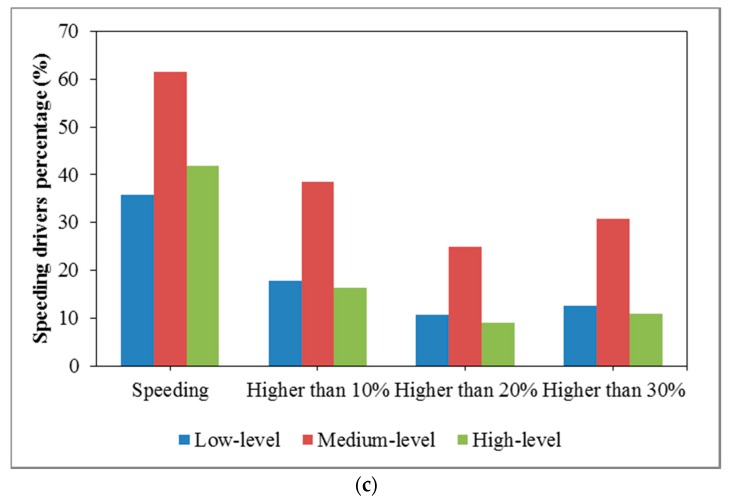
Speeding ratio distributions in terms of scenario, gender and driving experience. (**a**) Countermeasures; (**b**) gender; (**c**) driving experience.

In order to analyze the effectiveness of the countermeasures, the speeding ratio is another important measure that reflects the drivers’ speeding behaviors in general. Figure 3 shows the speeding ratios at the point of the city entrance with different factors, including countermeasures, gender and driving experience. From Figure 3, the speeding ratio has a clear downward trend when any countermeasure is applied in the scenario. In addition, compared to the male drivers, female drivers have a lower speeding ratio at all different speeding levels. The findings are consistent with the previous study results done by Teo et al. [43]. For the driving experience, the medium-level group has the highest speeding ratio at all levels. There is no obvious difference between the low-level group and the high-level group.

Logistic regression analysis was applied to investigate the impacts of potential influencing factors on the incidence of the speeding at the city entrance, as shown in Table 3. The independent variables include countermeasures, gender and the driving experience. The baseline, the male and the low-level driving experience group are treated as the reference categories. The previous studies indicated that it was much more dangerous when drivers were speeding over 20% of the speed limit [44,45]. Therefore, the dependent variable is speeding behavior at the city entrance, which was classified into two levels: speeding less than 20% of the speed limit and speeding over 20% of the speed limit. Based on the logistic regression model, countermeasures and driving experience are significantly associated with speeding behavior based on a 0.1 significance level, whereas gender does not show significant effects on the incidence of the speeding behavior at the city entrance. 

For the effect of the driving experience, drivers with medium-level experience are more likely to have speeding behaviors than the other two levels. The speeding over 20% of the speed limit occurrence likelihood of the drivers with medium-level experience is nearly three-times (Exp(B) = 2.916) that of the drivers with low-level experience. However, drivers with high-level driving experience have no significant difference in speeding occurrence compared to the low-level driving experience group. It is widely acknowledged that young novice drivers have higher accident risk than experienced drivers, which is mainly because of the lack of driving skills [46,47,48,49,50]. Therefore, they could not deal with the complex road environment and traffic situations. However, in this study, the road network is just a simple straight road, and no other vehicles show up in the transition zone, so driving skills are not the problem for novice drivers. Therefore, the cautious psychological state of novice drivers makes them have a lower speeding ratio. In comparison, the veteran drivers have rich driving experience, so that they have better performance on speeding behavior. However, drivers with medium-level driving experience without both a cautious psychology and rich driving experience may be the group who is more likely to speed.

As for the countermeasures, the scenarios with the in-vehicle audio warning system have lower speeding occurrence likelihood (*p* = 0.022) than the baseline scenario based on the 0.05 significance level, which means that the audio warning system has effects on helping drivers reduce the speeding ratio when they enter into the city. However, the lit speed limit scenario and the combination of audio warning system and lighting scenario have the same, but not as strong as the audio warning system, effective level on decreasing the speeding ratio (lighting: *p* = 0.086; lighting + audio warning: *p* = 0.086). The possible reason is that after drivers see the lit reminder speed limit sign, they would not care about the audio warning system any more. Therefore, the audio warning system only is the best countermeasure based on the analysis of the speeding ratio.

### 3.3. Deceleration Performance Analysis

**Figure 4:**
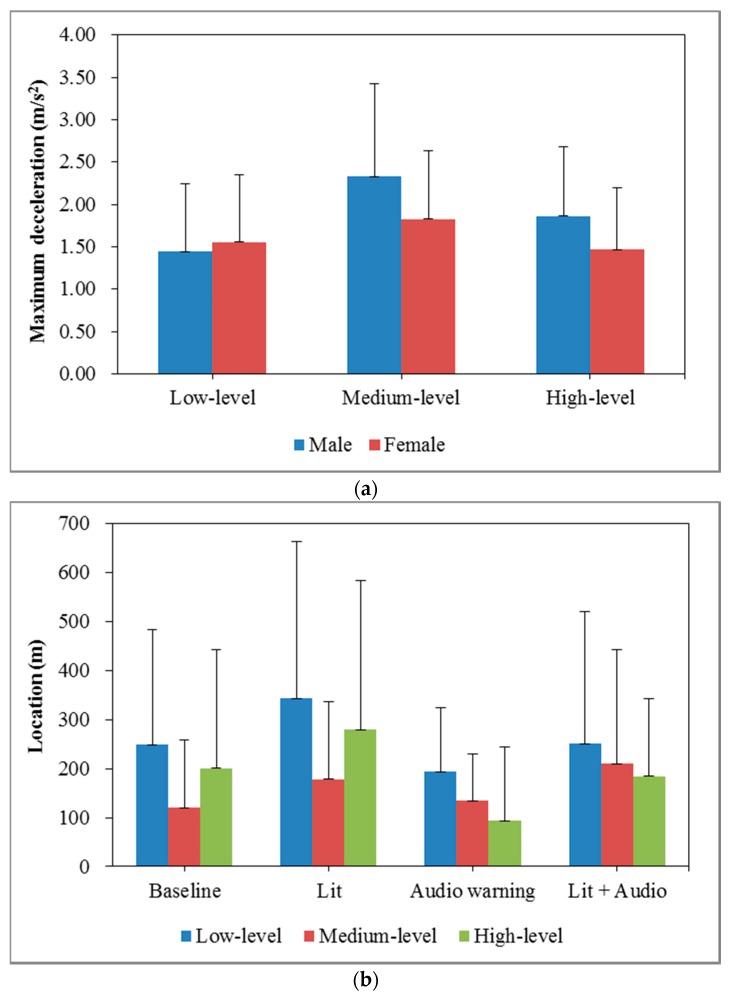

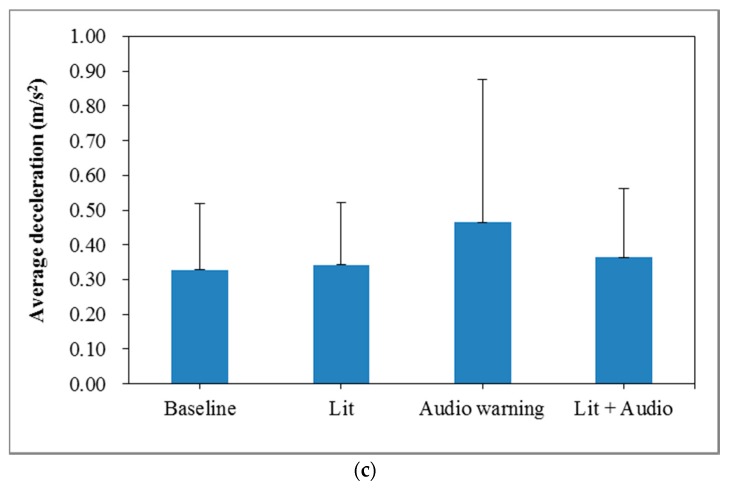
Maximum deceleration, corresponding location and average deceleration with significant factors. (**a**) Maximum deceleration with driving experience and gender; (**b**) location with scenario and driving experience; (**c**) average deceleration with scenario.

The measures of maximum deceleration, location and the average deceleration are used for exploring how the different countermeasures affect the driving behavior during the deceleration period. In this study, the multivariate analysis of variance (MANOVA) is conducted to investigate the effect of potential factors on maximum deceleration, location and the average deceleration during the deceleration period. The results indicate that there is no significant difference in maximum deceleration among all countermeasures (baseline: M = 5.36 m/s^2^, SD = 3.02 m/s^2^, lighting: M = 6.34 m/s^2^, SD = 3.38 m/s^2^, audio warning: M = 6.95 m/s^2^, SD = 3.24 m/s^2^, lighting + audio warning: M = 6.52 m/s^2^, SD = 3.13 m/s^2^; *p* = 0.138). However, gender and driving experience have significant effects on the maximum deceleration (male: M = 6.77 m/s^2^, SD = 3.49 m/s^2^; female: M = 5.80 m/s^2^, SD = 2.81 m/s^2^; *p* = 0.053; low-level experience: M = 5.40 m/s^2^, SD = 2.84 m/s^2^; medium-level experience: M = 7.24 m/s^2^, SD = 3.56 m/s^2^; high-level experience: M = 6.01 m/s^2^, SD = 2.84 m/s^2^; *p* = 0.002), as shown in Figure 4. The result implies that male drivers have a higher maximum deceleration than female drivers, and the drivers with medium-level driving experience have the highest maximum deceleration. Combined with the fact that female drivers are less likely to have speeding behaviors at the city entrance above, it is clear that the female drivers would have stable deceleration, while the male drivers tend to have hard brakes. Previous research also indicated that male drivers were deemed to engage in the hard brake behavior on the road [51,52,53]. Moreover, driving experience (*p* = 0.044) and the different countermeasures (*p* = 0.073) have significant effects on the location. From Figure 4, the drivers with low-level driving experience decelerate earlier than those with the medium-level and high-level driving experience when they enter the transition zone. In addition, the results also display that the location of the maximum deceleration in the scenario with the lit speed limit sign only might be far from the city entrance, which indicates the lit reminder could make drivers decelerate earlier than the other three situations. 

Further, the MANOVA test result for the average deceleration indicates that there is no significant difference between male and female drivers (male: M = 0.4 m/s^2^, SD = 0.32 m/s^2^, female: M = 0.35 m/s^2^, SD = 0.18 m/s^2^; *p* = 0.189) and among the driving experience groups (low-level experience: M = 0.35 m/s^2^, SD = 0.18 m/s^2^; medium-level experience: M = 0.43 m/s^2^, SD = 0.36 m/s^2^; high-level experience: M = 0.35 m/s^2^, SD = 0.21 m/s^2^, *p* = 0.172). However, it is found that the average deceleration is marginally significantly associated with the countermeasures (*p* = 0.086), which is also shown in Figure 4. It is found that the scenario with the audio warning system only has the highest average deceleration compared to the other three scenarios.

## 4. Discussion

The purpose of this study is to investigate the impacts of the in-vehicle audio speeding warning message system on drivers’ speed performance in transition zones based on driving simulator experiments. The experiment results showed that the audio warning system could significantly reduce driver’s operating speed before they entered the city. According to the logistic regression analysis, it was found that only the audio warning system could significantly reduce the probability of speeding over 20% of the speed limit based on a 0.05 significance level. However, the lit reminder scenario and the combination of audio warning system and lit speed limit sign scenario have the same, but not as strong as the audio warning system, effective level on decreasing the speeding behavior. The possible reason is that after drivers see the lit speed limit sign, they would not care about the audio warning system any more. As for the deceleration period, the countermeasure of the lit speed limit sign could make drivers slow down earlier compared to the other two countermeasures. The reason is that drivers could see the lit sign earlier than they hear the audio warning message. However, drivers would have a higher average deceleration when implementing the audio warning system. Overall, the lit sign has minimum positive effects on reducing the driver’s speed, while the implementation of the audio warning system plays an essential role in improving drivers’ speed control performance in the transition zone. 

Additionally, the results also indicate that male drivers have a higher operating speed and maximum deceleration than female drivers, and the drivers with medium-level driving experience have the highest operating speed in the transition zone. Previous researchers also found that inexperienced drivers were more likely to speed compared to experienced drivers [54,55], and they were less sensitive to the potential risk and had more speed-related collisions [49,56,57]. However, this paper categorized the group by driving experience and found that the medium-level driving experience drivers are more likely to speed. 

It should be noted that there exist several limitations in this research that are suggested for further studies. This paper is based on the experimental design to test the effects of different countermeasures in the speed transition zone and the patterns associated with different types of drivers. Without a large-scale sampling analysis, the impacts of drivers’ demographic factors cannot be identified in this study. Secondly, although the driving simulator is an effective tool for driving performance comparison studies, there are absolute validity issues in the driving simulators from the aspects of physical, perceptual and behavioral fidelity. Thus, a field test is recommended for validating the results from this study. Furthermore, only one road scenario was designed and tested in this experiment under a sunny daytime weather condition. More road types, weather features and different visibility conditions need to be studied in the future.

## 5. Conclusions

In this study, the effectiveness of traffic calming measures in the transition zone, which include lit speed limit sign only, audio warning system only and the combination of lit speed limit sign and audio warning system, was tested in the driving simulation experiment. According to the experiment results, the audio warning system could more effectively reduce drivers’ operating speed before they entered the city compared to the lit speed limit sign. Only the audio warning system could significantly reduce the probability of speeding at the city entrance. The countermeasure of the lit speed limit sign could make drivers slow down earlier, while drivers would have a higher average deceleration when implementing the audio warning system. In conclusion, the implementation of the in-vehicle audio speeding warning message system plays an essential role in improving drivers’ speed control performance in the transition zone from rural areas to urban areas.

## Figures and Tables

**Table 1:** Descriptive statistical results for average operating speeds for different scenarios, genders and driving experiences at different locations.

Location	Scenario								Gender				Driving Experience					
	Baseline		Lighting		Audio Warning		Lighting + Audio		Male		Female		Low-Level		Medium-Level		High-Level	
	Mean	SD	Mean	SD	Mean	SD	Mean	SD	Mean	SD	Mean	SD	Mean	SD	Mean	SD	Mean	SD
−700 m	73.04	11.55	72.83	7.99	73.79	8.24	72.11	7.48	74.31	8.87	71.48	8.71	71.16	7.37	75.53	8.69	72.31	9.94
−600 m	72.25	11.95	71.86	8.67	73.39	8.77	70.98	9.01	73.59	9.46	70.55	9.61	69.91	8.30	74.83	9.16	71.80	10.75
−500 m	71.02	12.27	69.95	9.37	72.99	8.85	69.70	9.64	72.12	10.15	69.63	9.94	68.83	9.18	73.54	10.03	70.55	10.63
−400 m	68.54	13.18	65.88	11.71	70.55	9.47	67.43	10.94	69.08	11.41	67.05	11.40	65.84	10.60	71.04	11.88	67.61	11.36
−300 m	65.28	14.32	61.98	12.36	67.92	10.86	64.62	11.06	65.87	12.31	63.96	12.27	62.25	11.85	68.32	12.20	64.49	12.27
−200 m	59.59	15.83	55.48	14.70	61.55	14.61	58.79	10.81	58.87	14.78	58.83	13.53	57.82	13.68	61.71	15.54	57.20	13.03
−100 m	50.16	14.28	47.19	12.14	52.01	14.98	48.19	11.18	49.83	13.12	48.91	13.42	48.66	12.98	51.10	14.92	48.50	11.84
0 m	46.01	12.18	43.25	9.43	43.91	11.40	41.41	7.35	44.09	9.53	43.14	11.07	41.11	10.32	46.26	11.20	43.70	8.80
100 m	45.21	10.67	42.59	8.01	42.71	8.11	40.83	5.72	44.13	8.63	41.43	7.90	39.98	8.09	46.22	8.35	42.49	7.61
200 m	45.14	10.52	42.55	8.46	41.77	5.83	40.78	6.55	44.02	8.97	40.98	6.82	40.53	6.48	45.65	9.62	41.65	7.28
300 m	44.60	10.58	42.43	8.81	40.34	5.89	40.66	7.95	43.40	9.55	40.49	7.11	39.86	6.51	45.23	10.22	41.09	7.86

**Table 2:** The paired *t*-test results for operating speed at each location.

Location	Baseline vs. Lighting			Baseline vs. Audio Warning			Baseline vs. Lighting + Audio		
	*t*	df	*p*-value	*t*	df	Sig.	*t*	df	*p*-value
−700 m	0.207	39	0.837	−0.385	39	0.702	0.773	39	0.444
−600 m	0.324	39	0.748	−0.585	39	0.562	0.940	39	0.353
−500 m	0.712	39	0.481	−0.980	39	0.333	0.906	39	0.371
−400 m	1.341	39	0.188	−0.957	39	0.344	0.691	39	0.494
−300 m	1.542	39	0.131	−1.138	39	0.262	0.445	39	0.658
−200 m	1.672	39	0.102	−0.613	39	0.544	0.533	39	0.597
−100 m	1.510	39	0.139	−0.706	39	0.485	1.152	39	0.256
0 m	1.925	39	0.062	1.036	39	0.307	3.379	39	0.002 *
100 m	1.597	39	0.118	1.516	39	0.137	3.185	39	0.003 *
200 m	1.616	39	0.114	2.631	39	0.012 *	3.533	39	0.001 *
300 m	1.344	39	0.187	3.502	39	0.001 *	3.427	39	0.001 *
400 m	1.399	39	0.170	2.626	39	0.012 *	2.314	39	0.026 *

* Indicates that the variable is significant at the 95% confidence level.

**Table 3:** Parameter estimates of logistic regression models for the speeding behavior.

Variable	Level	B	SE	Wald	df	Sig.	Exp(B)
Scenario	Baseline vs. Light reminder	−1.054	0.614	2.949	1	0.086	0.349
	Baseline vs. Audio warning	−1.641	0.714	5.274	1	0.022	0.194
	Baseline vs. Light + Audio	−1.054	0.614	2.949	1	0.086	0.349
Gender	Male vs. Female	−0.529	0.476	1.234	1	0.267	0.589
Driving Experience	Low-level vs. Medium-level	1.070	0.556	3.703	1	0.054	2.916
	Low-level vs. High-level	−0.179	0.652	0.075	1	0.784	0.836
Constant			−1.086	0.553	3.854	1	0.050
